# Economic impact of COVID-19 on patients with type 2 diabetes in Kenya and Tanzania: a costing analysis

**DOI:** 10.1136/bmjph-2023-000383

**Published:** 2024-08-24

**Authors:** Caroline H Karugu, Peter Binyaruka, Patrick G Ilboudo, Richard E Sanya, Shukri F Mohamed, Lyagamula Kisia, Peter Kibe, Irene Mashiashi, Christopher Bunn, F Mair, Charles Agyemang, Sally M Mtenga, Gershim Asiki, Cindy M Gray, Eleanor Grieve, Manuela Deidda

**Affiliations:** 1 Chronic Diseases Management, African Population and Health Research Center, Nairobi, Kenya; 2 Public and Occupation Health, Amsterdam Medical Center, University of Amsterdam, Amsterdam, Noord-Holland, The Netherlands; 3 Ifakara Health Institute, Dar es Salaam, Tanzania; 4 Maternal and Child Wellbeing Unit, African Population and Health Research Center, Nairobi, Kenya; 5 Institute of Public Health, University of Glasgow College of Social Sciences, Glasgow, UK; 6 General Practice and Primary Care, University of Glasgow, Glasgow, UK; 7 Department of Health Systems, Impact Evaluation and Policy, Ifakara Health Institute, Dar es Salaam, Tanzania; 8 Health and well-being- Chronic disease management, African Population and Health Research Center, Nairobi, Kenya; 9 School of Social and Political Sciences, University of Glasgow, Glasgow, UK; 10 Institute of Health and Wellbeing, University of Glasgow College of Medical Veterinary and Life Sciences, Glasgow, UK

**Keywords:** COVID-19, Public Health, economics, Cross-Sectional Studies

## Abstract

**Introduction:**

COVID-19 affected healthcare access, utilisation and affordability, especially for patients suffering from chronic diseases, including type 2 diabetes (T2D). This study measured the occurrence and magnitude of changes in healthcare and broader societal costs among patients with T2D before and during COVID-19 in Kenya and Tanzania to understand whether and how COVID-19 affected T2D management in countries implementing different policies during the pandemic.

**Methods:**

A cross-sectional study was conducted in Kenya and Tanzania in March–April 2022 among 500 patients with T2D in each country. We interviewed patients on direct healthcare costs (eg, inpatient and outpatient costs), societal costs (eg, productivity loss) and patients’ characteristics before and during the COVID-19 pandemic. We estimated changes over time using the Generalised Linear Model in Kenya and a two-part model in Tanzania, adjusting for patient-level covariates.

**Results:**

The overall costs of management of T2D in most categories increased in both countries during COVID-19, but some of the increase was not significant. Transport and testing costs increased significantly in Tanzania (I$0.33, p<0.01 and I$0.85, p<0.01) but not in Kenya (I$1.69, p=0.659 and I$0.10, p=0.603). Outpatient costs increased significantly in Tanzania (I$8.84, p<0.01) but there was no significant change in Kenya (I$8.09, p=0.432). T2D medication costs did not change in Tanzania (I$0.19, p=0.197), but decreased significantly in Kenya (I$18.48, p<0.01). Productivity losses increased significantly in both countries.

**Conclusion:**

The COVID-19 pandemic is associated with increased direct costs but with a significant increase in many cost categories (transport, testing and outpatient) in Tanzania than in Kenya. A significant increase in productivity loss was observed in both countries. The minimal cost increases in Kenya may be due to the inaccessibility of services associated with lockdown measures and higher insurance coverage compared with Tanzania. Pandemic preparedness initiatives and interventions are needed to safeguard the welfare of patients with chronic conditions during pandemics.

**WHAT IS ALREADY KNOWN ON THIS TOPIC**

- COVID-19 adversely affected people with underlying conditions such as non-communicable diseases (NCDs), including type 2 diabetes (T2D).
- COVID-19 affected healthcare access, costs, utilisation and affordability, especially for patients suffering from chronic diseases like T2D.

**WHAT THIS STUDY ADDS**

- This paper shows that the total direct and indirect costs increased slightly during COVID-19 in Kenya and Tanzania, but the individual cost categorisations showed varying increases and decreases in the marginal costs.
- A breakdown by subcategories revealed similarities in broader social costs (loss of productivity) but differences regarding outpatient visits and glucose testing by country.

**HOW THIS STUDY MIGHT AFFECT RESEARCH, PRACTICE OR POLICY**

- It shows healthcare costs with different patterns in the two countries due to the differences in COVID-19 restrictions and policies, but the societal impact was similar.
- There is a need for continued efforts to develop targeted strategies for the management of T2D during epidemics.
- Policymakers need to extend policies to other chronic diseases in order to ensure patients’ capacity to self-manage T2D and other chronic diseases effectively during any future pandemic.

## Background

 Diabetes is among the top 10 causes of death globally.^1^ According to estimates by the International Diabetes Federation, in 2021, 537 million people had diabetes, and 6.7 million deaths were caused by diabetes worldwide.^2^ It is estimated that the global prevalence of diabetes will increase to 647 million and 783 million by 2030 and 2045, respectively.^2^ The increase in prevalence is disproportionately higher in low- and middle-income countries (LMICs) than in high-income countries due to an increase in overweight/obesity (high body mass index (BMI)) and widespread physical inactivity.^3 4^ Currently, in sub-Saharan Africa (SSA), 24 million people have diabetes, and it has been estimated that the prevalence will increase to 55 million by 2045, a 134% increase and the highest rise compared with other regions worldwide.^2^ East African countries such as Kenya and Tanzania are no exception. The prevalence of diabetes among adults in Kenya was 2.4% in 2015 and more recent projections estimate a 3% prevalence.^2^ In Tanzania, 12.3% of the adult population have diabetes, the highest prevalence in SSA.^2^ Non-communicable diseases (NCDs), including type 2 diabetes mellitus (T2D), have posed a significant economic burden in SSA, both at the individual household level and to society and the public healthcare system.^5^ However, variances could be noted in cost drivers due largely to differences in cost perspectives (patient vs societal). For instance, the annual direct costs of management of T2D in Africa ranged from I$ 3.5 billion to 4.5 billion between the countries, with indirect costs being higher than the direct costs per patient.^5^ The total economic costs of managing diabetes in Kenya in 2019 were estimated to be $372 184 585, with an annual cost per patient of $674, where the total direct costs were the key drivers of the costs, accounting for 61% of the costs.^6^ T2D accounts for more than 90% of all diabetes cases and is therefore the main cost driver for diabetes management.^2^

Many LMICs, including Kenya and Tanzania, face significant healthcare resource constraints, health workforce shortfalls and fragmented care delivery for diabetes.^7^,^9^ This was worsened by the COVID-19 pandemic in early 2020, for various reasons.^10^ Healthcare costs in Kenya and Tanzania are financed by taxation, donor support, health insurance schemes and out-of-pocket costs, which were severely constrained by the COVID-19 pandemic.^11 12^ The limited health workforce and related health resources for routine chronic disease management, including T2D, were often diverted for emergency response.^13^ Also, individuals with T2D had an increased risk of severe COVID-19, adding further economic strain to patients with diabetes and the health system.^14^,^18^ COVID-19-related travel restrictions and ‘lockdown’ measures instituted by some governments to control the pandemic had economic consequences for individuals and households.^19^ This was largely due to the household-level economic strife in LMICs and widespread unemployment or irregular access to gainful employment during the COVID-19 pandemic.^20 21^

At the start and peak of the pandemic, the governments of Kenya and Tanzania deployed different approaches to control the pandemic. Kenya instituted strict lockdowns and curfews with enforcement of COVID-19 testing at border points,^22^ while Tanzania used a more relaxed approach with no ‘lockdown’ measures.^23^ However, a disruption of income-generating activities was reported in both countries.^24^

In SSA, there is scarce evidence on the impact of economic disruption induced by the COVID-19 pandemic on the healthcare and societal burden experienced by patients with T2D. In this paper, we investigated the occurrence and magnitude of changes in healthcare costs (testing, hospitalisation and outpatient visits) and broader social costs (loss of productivity, formal and informal care), before and during the peak of the COVID-19 pandemic, among patients with T2D in Kenya and Tanzania. Results will inform policy and practice initiatives to make the healthcare and social systems supportive to people with chronic conditions such as T2D during pandemic situations or any other state of emergency.

## Methods

The study used a cross-sectional survey conducted in Kenya and Tanzania among 500 patients with T2D in each country. Participants were asked to report healthcare direct and indirect resource use before and during the COVID-19 pandemic to explore whether any change in health-seeking patterns, healthcare costs or socioeconomic conditions arose due to the pandemic.

### Study sites and sampling

The study was conducted in four counties in Kenya: Nairobi (n=276), Kiambu (n=104), Vihiga (n=76) and Nyeri (n=44). and two regions in Tanzania: Dar es Salaam (n=300) and Morogoro (n=200), among adults diagnosed with T2D before COVID-19 (ie, March 2020). Kenya and Tanzania, both LMICs were purposively selected based on the notable differentiated prevalence of diabetes mellitus and the varied responses to COVID-19 measures and restrictions. As of August 2023, Tanzania had reported 43 078 confirmed COVID-19 cases and 846 related deaths, while Kenya had recorded 343 918 confirmed cases and 5689 deaths attributed to COVID-19.^25^ During the study period, the COVID pandemic was not at its peak, but we purposively intended to assess the cost implications of managing T2D in the most disrupted months during the pandemic. We estimated the sample size for the two countries using the Cochran formula below^26^:

$$n_{0}=\frac{Z^{2}*p*\left(1-p\right)}{e^{2}}$$

We assumed p=0.5, representing 50% of patients with T2D experiencing disruption of care during COVID-19 based on the WHO report (Z=1.96), considering a power of 80% at a 95% CI, and a margin of error (e=0.05) representing a 5% acceptable sampling error.^10 27^ Substituting in the formula above provides a sample size of 384, which we increased by 30% to cater to the non-response rate assumption, thus giving a total sample size of 499 corrected to 500 in each country. Patients aged 18 years and above, those diagnosed with T2D and other comorbidities and those who were receiving care for T2D before and during the COVID-19 pandemic were eligible to participate in this study. Eligible patients were identified from health facility patient registers at the outpatient departments. All eligible patients were approached, and written informed consent for Tanzania and, in Kenya, verbal recorded consent was obtained.

### Data collection

A survey questionnaire was administered to capture the healthcare and societal costs and consequences associated with T2D, pre and during the worst or most disrupted months of receiving care during the COVID-19 pandemic. The questionnaire used in this study has been attached as online supplemental file 1. A visual socioeconomic status (SES) ladder was used for comparability between countries. Trained fieldworkers administered structured questionnaires in both countries. The tool was piloted and refined before use. Data collection in both countries was conducted from February to April 2022. Tanzania’s data collection used face-to-face interviews with eligible patients, while Kenya’s data collection used phone interviews for the survey due to COVID-19 lockdown measures in place. This study was approved by the Ethics and Scientific Review Committee at AMREF Health Africa in Kenya (ESRC P900-2020) and the National Institute for Medical Research (NIMR/HQ/R.8a/Vol.IX/3806) in Tanzania. The participants were required to sign informed consent forms prior to participating in the study.

### Patient and public involvement

The survey questionnaire was developed in consultation with local stakeholders. Participants with T2D were recruited at the health facilities with the support of the healthcare providers in charge of the health facilities. With the consent and permission from patients and those in charge of the health facilities, participants’ medical records were consulted to identify participants’ comorbid conditions. The preliminary findings were disseminated to patients, healthcare providers, community health volunteers and other stakeholders, and participatory workshops were held to understand the stakeholders’ views of the outcomes of the study. All these groups, starting from the community level, including patients, healthcare providers and the government and policymakers, contributed to the patient and community level and nationally representative-related recommendations to the policymakers for improving T2D care at the community level.

### Resource use and unit costs

#### Direct costs

The cost of healthcare, medication, tests and transportation was self-reported by participants. Outpatient visits and hospitalisations were valued using National Health Insurance Fund (NIHF) unit costs for Tanzania,^28^ and WHO unit costs for Kenya^29^ (table 1). WHO costs are categorised into primary, secondary and tertiary-level facilities, and an average unit cost was applied. For Tanzania, we used the unit costs from hospitals, where the cost by the type of health facility by ownership (government, faith-based organisations and private hospitals) was collected (rather than level).

**Table 1:** Unit cost estimation

Item	Unit cost (local currency)	Value adjusted by inflation in 2021	Source+year	Unit cost I$*†	Assumptions/notes
Kenya					
Hospitalisation	374.51	200.98	WHO costing tool, 2011	4.59	Outpatient visit cost: WHO estimation, sheet prices table cost per outpatient visit to a health centre, assume a coverage level of 50% and average duration of visit of 40 min
Outpatient visits	142.12	76.27	WHO costing tool, 2011	1.74	Hospital visits and stays: assume visits/stays in primary hospital
Tanzania					
Hospitalisation-public	72 028.82	62 805.62	2017/2018	70.52	All unit costs from Tanzania National Health Insurance Fund (NHIF, 2017/2018)
Hospitalisation-private	233 301.38	203 427.44	2017/2018	228.42	
Hospitalisation-FBO/NGO	94 524.11	82 420.42	2017/2018	92.55	
Outpatient-public	15 488.67	13 505.37	2017/2018	15.16	
Outpatient-private	24 738.01	21 570.34	2017/2018	24.22	
Outpatient-FBO/NGO	19 569.54	17 063.69	2017/2018	19.16	

* Inflation to 2021 was applied using the Consumer Price Index (source: World Development Indicators).

† Converted to international dollars (I$) using TZ (890.60) and Kenya (43.80) PPP 2021; source: World Development Indicators.

#### Indirect costs

Loss of productivity (absenteeism) cost was measured as the number of days lost because of health reasons related to diabetes, pre-COVID and during COVID-19, and valued using average (monthly) wage.^30^ The cost of formal care was self-reported, while the opportunity cost of informal care was measured as the number of days a (unpaid) family member takes care of the individual because of his/her ill health, multiplied by country-specific average wage. All productivity and formal and informal care costs were scaled to 1 year.

All direct and indirect costs were adjusted to 2021 inflation and converted to international dollars using the purchasing power parity conversion rate for each country.^31^

### Data analysis

Descriptive summaries were used to explore direct and indirect costs before and during the COVID-19 pandemic in both countries. Standard regression models were fitted with several cost categories (overall direct costs, healthcare costs, transport costs, testing costs, medication costs and inpatient and outpatient costs) as the dependent variable. Cost variables were regressed against the time dummy indicator and other covariates, including sociodemographic (location, gender, marital status, age, education level, religion, occupation, insurance status and household socioeconomic status) and health-related variables (family history of T2D, time living with T2D and number of comorbidities).

Although the data were collected at only one point in time, we accounted for potential correlation within individuals reporting on two time points by adjusting regression models for clustering. For Kenya, a clustered Generalised Linear Model (GLM) gamma regression was run, adjusting for individual random effects. The model allows for functional forms that account for non-normality with GLM/gamma log used and the skewed costs. For Tanzania, a two-part model was run to account for the large proportion of zero-cost data in this country.^32^ The first part estimated a logit regression to determine the likelihood of incurring costs, and a GLM model with SE adjustment for clustering was fitted in the second part. For impact on productivity, formal and informal care, a two-part model was used to analyse the costs incurred in the large number of zero-cost data in both countries. STATA software (V.15) was used for data analysis.

## Results

### Characteristics of patients recruited in the study

The demographic characteristics of the patients with T2D in both countries are shown in table 2. The majority of the participants in both countries were female, living in urban areas, married, self-employed, had a family history of diabetes, had lived with diabetes for more than 6 years, had at least one other comorbidity and belonged to a lower SES level. Most participants had attained primary and secondary education and had self-employment in small and large businesses. The mean (SD) age in Kenya and Tanzania was 58.2 (12.6) and 56.8 (10.2), respectively. We found the majority of the participants from Kenya to have health insurance coverage (67.4%), whereas only 39.6% from Tanzania had health insurance coverage.

**Table 2:** Demographic characteristics and summary descriptive costs of patients with T2D in Kenya and Tanzania

Variable	Categories	Kenya (n=500)	Tanzania (n=500)
		n (%)	n (%)
Region	Urban	380 (76%)	300 (60.0%)
	Rural	120 (24%)	200 (40.0%)
Sex	Female	330 (66%)	336 (67.2%)
	Male	170 (34%)	164 (32.8%)
Age in years, mean (SD)	All	58.2 (12.6)	56.8 (10.2)
Age group	<40 years	39 (7.8%)	36 (7.2%)
	40–49 years	80 (16%)	62 (12.4%)
	50–59 years	135 (27%)	165 (33%)
	60–69 years	155 (31%)	206 (41.2%)
	>70 years	91 (18.2%)	31 (6.2%)
Formal education	None	16 (3.2%)	34 (6.8%)
	Primary	208 (41.6%)	298 (59.6%)
	Secondary	201 (40.2%)	126 (25.2%)
	College/University	75 (15%)	42 (8.4%)
Marital status	Married	319 (63.8%)	312 (62.4%)
	Not married	181 (36.2%)	188 (37.6%)
Religion	Catholics	104 (20.8%)	137 (27.4%)
	Protestants	379 (75.8%)	110 (22%)
	Muslims	17 (3.4%)	253 (50.6%)
Occupation	Formal employment	31 (6.2%)	40 (8%)
	Farming (small and large scale)	78 (15.6%)	103 (20.6%)
	Self-employed (small and large business)	162 (32.4%)	190 (38%)
	Homemaker (housewife/husband)	14 (2.8%)	0
	Retired	55 (11%)	72 (14.4%)
	Unemployed	160 (32%)	95 (19%)
Family history of diabetes	No	233 (46.6%)	205 (41%)
	Yes	267 (53.4%)	295 (59%)
Duration from first diagnosis of type 2 diabetes	>6 years	309 (61.8%)	334 (66.8%)
	<6 years	191 (38.2%)	166 (33.2%)
Health insurance coverage	Uninsured	163 (32.6%)	302 (60.4%)
	Insured	337 (67.4%)	198 (39.6%)
Presence of comorbidities	Yes	355 (71%)	368 (73.6%)
	No	141 (29%)	132 (26.4%)
Economic status (ladder scale (1–10))	Lower SES (1–5)	447 (89.4%)	374 (74.8%)
	Higher SES (6–10)	53 (10.6%)	126 (25.2%)
Direct costs during COVIDmean (SD)	Total direct costs	120.29 (154.13)	19.33 (27.16)
	Healthcare costs	106.79 (143.81)	15.45 (26.27)
	Transport costs	13.50 (27.74)	3.88 (6.09)
	Testing costs	23.85 (49.62)	4.27 (8.94)
	Medication costs	76.37 (223.11)	14.89 (26.86)
	Hospitalisation costs	478.67 (10700.49)	19.26 (55.94)
	Outpatient costs	66.90 (261.82)	47.89 (76.04)
Indirect costs during COVIDmean (SD)	Total costs	19 443.51 (7203.49)	3359.28 (2630.28)
	Productivity	6471.88 (7172.45)	1155.13 (1783.27)
	Informal care	13 009 (1364.46)	1702.58 (1500.25)

Summary descriptive statistics of the costs incurred by the patients before and during the COVID-19 pandemic are provided in table 2 (see online supplemental appendix A for full details). The average direct costs for managing T2D during the COVID-19 pandemic were I$19.33 and I$120.29 in Tanzania and Kenya, respectively. Similarly, the overall healthcare costs incurred were relatively higher in Kenya (I$106.79) compared with Tanzania (I$ 15.45), and this pattern was consistent across other cost categories on average, including indirect costs.

### Effect of COVID-19 on direct costs in Kenya and Tanzania

The predicted costs, pre-COVID-19 and during COVID-19, and the marginal effects, as well as the time variable coefficient and associated value from the GLM regression (ie, its magnitude and significance) for each direct cost category are reported in table 3. The results show that COVID-19 was associated with a significant increase in the amount paid on average for transport, testing and outpatient care in Tanzania and associated significantly with a decline in medication costs in Kenya (table 3). Hospitalisation costs in Tanzania increased non-significantly by I$2.70 but decreased in Kenya non-significantly by I$3.60 during the COVID-19 period. All the remaining cost categories did not change significantly. Full regression outputs for each direct cost category in both countries are provided in online supplemental appendix B1 (Tanzania) and online supplemental appendix B2 (Kenya). The logit results in online supplemental appendix C show a reduced likelihood of incurring costs for healthcare, transport and medication during the COVID-19 pandemic in Tanzania, while the chances of incurring hospitalisation costs increased significantly.

**Table 3:** Effect of COVID-19 on direct costs in Kenya and Tanzania: predicted costs pre/post+marginal effects

	Healthcare cost	Transport cost	Total direct cost	Testing cost	Medication cost	Hospitalisation cost	Outpatient cost
Tanzania							
Predicted pre-COVID costs (I$) (SE)	16.07(0.99)	3.52(0.21)	20.45(1.25)	3.51(0.38)	15.05(0.84)	17.07(2.74)	36.16(1.26)
Predicted during COVID costs (I$) (SE)	15.76(0.99)	3.85(0.24)	20.08(1.25)	4.35(0.45)	14.86(0.92)	19.78(2.48)	44.97(2.42)
Marginal costs COVID costs (I$) (SE)	–0.31(0.61)	0.33***(0.11)	–0.37(0.78)	0.85***(0.16)	–0.19(0.64)	2.70(3.28)	8.84***(2.11)
Time variable: coefficient (p value)	0.05(0.082)	0.106(0.000)	−0.004(0.912	0.22(0.000)	0.05(0.197)	−0.18(0.065)	0.21(0.000)
Number of observations	1000	1000	1000	1000	1000	1000	1000
Kenya							
Predicted pre-COVID costs (I$) (SE)	103.72(7.75)	12.11(1.14)	115.49(8.31)	3.21(0.09)	91.63(7.01)	6.15(3.66)	59.71(7.43)
Predicted during COVID costs (I$) (SE)	107.55(6.13)	13.80(1.20)	121.39(6.62)	3.30(0.16)	73.23(6.11)	2.55(1.41)	67.79(10.08)
Marginal COVID costs (I$) (SE)	3.83(8.61)	1.69(1.49)	5.91(9.16)	0.10(0.19)	−18.48*(7.22)	−3.60(2.69)	8.09(10.54)
Time variable: coefficient (p value)	0.04(0.659)	0.13(0.257)	0.05(0.522)	0.03(0.603)	−0.22*(0.010)	−0.88*(0.019)	0.13(0.432)
Number of observations	1000	1000	1000	1000	1000	1000	1000

Adjusted covariates include location, gender, marital status, age, education level, religion, occupation, insurance status, household socioeconomic status, family history of T2D, time living with T2D and number of comorbidities.

Coefficient+p value from the Generalised Linear Model.

### Effect of COVID-19 on indirect costs in Kenya and Tanzania

The predicted cost, pre-COVID-19 and during the COVID-19 pandemic and the marginal effect, as well as the time variable coefficient and its p value from the GLM (ie, its magnitude and significance) for the productivity loss costs in the two countries, are reported in table 4. The results show that COVID-19 had a significant and positive impact on both the likelihood of incurring productivity losses and the condition of having positive costs on their amount (table 4). Full regression outputs for the productivity loss costs in both countries, including the probability of incurring costs (logit) in Tanzania, are provided in online supplemental appendix D1 and D2. The analysis shows a significant increase in productivity loss costs in Tanzania and Kenya during the COVID period after adjusting for different sociodemographic and clinical factors. The regression analysis was not conducted on formal and informal care costs due to the high proportion of missing data.

**Table 4:** Effect of COVID-19 on indirect costs in Kenya and Tanzania: predicted costs pre/post+marginal effects

	Productivity
Tanzania	
Predicted pre-COVID costs (I$)	917.93
Predicted during COVID costs (I$)	1173.07
Time variable: coefficient, p value	0.122 (<0.01)
Number of observations	1000
Kenya	
Predicted pre-COVID costs (I$)	3347.77
Predicted during COVID costs (I$)	6433.64
Time variable: coefficient, p value	0.398 (<0.01)
Number of observations	936

1 Adjusted covariates include location, gender, marital status, age, education level, religion, occupation, insurance status, household socioeconomic status, family history of T2D, time living with T2D, and number of comorbidities.

Co-efficient + p value from the GLMGeneralised Linear M model.

## Discussion

The COVID-19 pandemic affected healthcare access and utilisation, especially among vulnerable populations, including people with chronic diseases like diabetes. Currently, there is little evidence of the cost burden imposed on patients with T2D by the COVID-19 pandemic in terms of healthcare and broader societal costs in LMICs. This study provides evidence on the cost of managing T2D before and during COVID-19 in Kenya and Tanzania, answering whether COVID-19 had a significant impact on healthcare and broader societal costs. The COVID-19 pandemic had an adverse impact on the socioeconomic situation of patients in the two countries, despite the total direct costs not being statistically different during the pandemic.

The overall costs of managing T2D in most categories increased in both countries during COVID-19, but some of the increase was not significant. Transport, testing and outpatient costs increased significantly in Tanzania but not in Kenya. T2D medication costs did not change in Tanzania but decreased significantly in Kenya. However, productivity losses increased significantly in both countries. We acknowledge the contextual differences between the two countries. We therefore attempted to compare the effects on costs but with close consideration of contextual differences. The differential effects on costs can be explained by three contextual reasons. First, the presence of lockdown measures in Kenya affected access to services, hence limited increases in costs, which is different from Tanzania.^33^,^35^ Second, insurance coverage was relatively higher in Kenya than Tanzania, as observed in this study, possibly explaining the limited cost escalation in Kenya compared to Tanzania. Third, the increase in testing costs in Tanzania is likely due to increased testing in private pharmacies and laboratories following a reduced utilisation of services and severe disruption of care in hospitals, which is evident in our data.^36^

However, when considering wider societal costs, productivity loss was significantly impacted during COVID-19 in both countries. This is possibly due to the fact that most patients reduced their mobility, avoiding crowded spaces, including workplaces, and allocated more time to improve and sustain their health. Because of the very low number of responses, it was not possible to explore whether COVID-19 was associated with any change in formal and informal care. The divergence of the findings in the two countries after adjusting for the clinical and demographic characteristics of patients with T2D in the two countries indicates the heterogeneity of the costs incurred in different settings of SSA.

Our findings are similar to those of other studies conducted among patients with T2D before and during COVID-19. A study conducted in Bangladesh showed increased glucose levels, complications and costs of care and disruption of access to care among patients with T2D during the COVID-19 pandemic.^37^ The decreased costs of diabetic medication during the COVID-19 period in Kenya are in contrast with another Kenyan study showing increased costs of insulin medication and reduced healthcare utilisation.^38^ Healthcare services and access were disrupted in Kenya during the pandemic, specifically the significant decline in the prescription of oral hypoglycaemics in Kenya, hence the overfall reduced costs.^36^ The reduction in utilisation and availability of T2D medication makes a strong case in our study, where the medication costs incurred were reduced significantly in Kenya and non-significantly in Tanzania during the COVID-19 pandemic. A study conducted in India on the implication of COVID-19 on the management of chronic conditions explained the loss of income as a driver for the inaccessibility of essential medication and job loss as significant predictors of depreciated diabetes symptoms.^39^ In other LMICs, the healthcare systems experienced severe disruptions in the provision of essential medical infrastructure that affected the service delivery and outcomes for patients with NCDs and other comorbidities.^40^ Similarly, in high-income countries, the burden of T2D during COVID was also experienced and there was great concern about the increased risks of mortality, morbidity and decreased economic productivity.^41^

The healthcare system was severely impacted during the COVID-19 pandemic, hence affecting service delivery to patients with T2D.^18^ This had a significant impact on costs based on the availability of healthcare services to patients in the countries. It was reported in other countries in Africa that lockdowns had an impact on glucose control, where 90.5% of patients had uncontrolled blood sugar during the lockdown, and this was higher than before the lockdown periods (82.9%).^42^ There was also reduced access to healthcare facilities and clinics, with reduced use frequency during lockdowns.^42^ In Tanzania, there were no lockdowns, but there was an increased surge of patients with respiratory conditions in the facilities.^23 35^ This evidence of the slight increases in costs in Kenya and Tanzania may be due to the inaccessibility of services and the lack of essential medication due to lockdowns, among other healthcare system constraints associated with the COVID-19 pandemic. In Kenya, like other LMICs, there was also a confirmed inability to access healthcare during lockdowns, hence reduced healthcare-seeking practices in urban slum areas.^43^ The differences in the costs can also be attributable to the different insurance mechanisms in Kenya and Tanzania, where the insured patients in Kenya experienced severe disruption, a reverse of what was observed in Tanzania.^36^ There was a significant decrease in overall outpatient visits in Kenya for patients with diabetes, showing a drop in healthcare system utilisation, which was similar to observations noted in other SSA countries.^44 45^ Healthcare system utilisation has an impact on the costs incurred by the patients in managing chronic conditions, implying that this might have economically affected the spending on the patients and the government. The overall disruption of the healthcare system in SSA, specifically in Kenya and Tanzania, indicates the implications and dynamics of a constrained environment for management and the economic utilisation and stability of patients with T2D.

### Strength and limitations

This study captured the different dynamics of costing in Kenya and Tanzania, which are different. This warranted the use of different approaches: a two-part model in Tanzania to account for the zero costs and the use of the generalised gamma regression model for Kenya. Furthermore, this is a unique study, as no patient-perspective economic analysis has been conducted in these countries. The results of this research are likely to form a blueprint for the formulation of country-specific recommendations that will contribute to the formulation of policies for diabetes management during pandemics.

However, this study has several limitations. The data were collected using a cross-sectional study design while recalling the experience before and during the COVID-19 period. This could have caused a potential recall bias because the costs were incurred at different periods for each person, which means the timescale of costs is less meaningful. However, this does not significantly affect the individual cost breakdowns, but the magnitude of predicted costs. In addition, we did not include whether someone had COVID-19 infection, which may have had a substantial impact on healthcare costs for the patient because less than 10% of the patients reported having tested positive for COVID-19 infection in both countries. The questionnaire did not include questions on food costs and accommodation costs; this could have been important considering that the diet for a patient with diabetes would cost more, and accommodation for those who travelled for specialised diabetes care had cost implications. We also did not capture T2D complications; instead, we used comorbidities. Furthermore, we expected an overestimation of productivity loss costs because patients with chronic illnesses and their caregivers were highly affected by the disruptions during the COVID-19 pandemic.

Furthermore, the sample was not drawn at random. Therefore, it may not be representative of the larger population, and the results may not apply to other groups or populations. Because of this, caution should be exercised when generalising the findings of this study to other settings. Finally, some assumptions on unit costs had to be made, as NHIF costs did not come at the same level of disaggregation of the resource use we collected. Although the sample was not randomly selected, the distribution of key variables like diversified geographical locations and gender in our sample resembles that of the general population. The costs shown in this study are an underestimation of the actual costs incurred by the patients and the public healthcare system because what is represented is an average of what is incurred in the three most disrupted months in 2021. The estimation of these costs per patient for the whole year, assuming that the patients incurred some costs in the other quarters of the year, would make the costs substantially higher. Therefore, despite the slight increase in economic costs of managing T2D in Kenya and Tanzania, there was a larger estimated cost.

## Conclusion

Overall, total costs increased slightly over time in both countries and a breakdown by subcategories reveals many differences. There were the same broader social costs (loss of productivity) in both countries, illustrating that the COVID-19 pandemic impacted patients’ management of diabetes, potentially leading to financial hardship. For Kenya, only the cost of medication reduced significantly, but other cost categories did not change significantly. For Tanzania, a significant increase in costs was observed in transport, testing and outpatient care. For wider societal costs, productivity loss significantly increased during COVID-19 in both countries. The results from this study give evidence of healthcare disruptions due to the COVID-19 pandemic but more specifically on healthcare costs and the management of chronically ill conditions. This calls for advocating for optimised healthcare systems with improved readiness to handle pandemics while incorporating essential care services for chronically ill patients in the SSA. It is high time for policymakers to ensure pandemic preparedness initiatives and interventions are implemented in order to safeguard the welfare of patients with chronic conditions during pandemics.

## supplementary material

10.1136/bmjph-2023-000383 online supplemental file 1

10.1136/bmjph-2023-000383 online supplemental file 2

## Data Availability

Data are available upon reasonable request.
