# Supplementary material for: Economic impact of COVID-19 on patients with type 2 diabetes in Kenya and Tanzania: a costing analysis
Source: BMJ Public Health. 2024 Aug 24;2(2):e000383. doi: 10.1136/bmjph-2023-000383 (PMC11816087; doi:10.1136/bmjph-2023-000383)
Supplement: online supplemental file 1 [file bmjph-2-2-s001.pdf]

SUPPLEMENTARY MATERIAL1: STUDY QUESTIONNAIRE

Healthcare and Socio-economic Impacts of COVID-19 on Patients with Diabetes in Kenya and

**1.**	Date: dd /mm /yyyy	
**2.**	Interviewer name:	
**3.**	Patient ID-number:	
**4.**	Name of health care facility	
**5.**	Are you suffering from Diabetes Type 2 condition?	
**6.**	How long you have been suffering from Diabetes Type 2 condition?	Years: _________________
**7.**	Apart from diabetes, do you have the following underlying conditions?	☐1 None<br>☐2 Hypertension / High Blood Pressure<br>☐3 Cardiac disease<br>☐4 Asthma<br>☐5 Chronic liver disease<br>☐6 chronic kidney disease<br>☐7 Chronic neurological/neuromuscular disease<br>☐8 COPD/chronic obstructive pulmonary disease<br>☐9 HIV/AIDs<br>☐10 Tuberculosis<br>☐11 Arthritis<br>☐12 Stomach/ bowel / other gastrointestinal problems<br>☐11 Depression/anxiety/schizophrenia<br>☐12 Cancer<br>☐13<br>Other……………………………………………
**8.**	Region	Kenya-(Nairobi, Kiambu, Nyeri, Vihiga)| Tanzania (Dar es salaam, Morogoro)
**9.**	County	
**10.**	Health facility name:	
**11.**	Facility ownership type:	☐1 Public<br>☐2 Private for profit<br>☐3 Private-NGO/faith based
**12.**	Facility Level of care	☐1 Hospital<br>☐2 Health Centre<br>☐3 Dispensary

Tanzania

**SECTION 1: SOCIO-DEMOGRAPHIC VARIABLES**		
**1.1**	Age	__________ years
**1.2**	Gender	☐ 1 Female ☐2 Male
**1.3**	Place of current residence	Village name ______________<br><br>☐ 1 Urban ☐2 Rural

**1.4**	Marital status	1. Married or living together<br>2. Divorced / separated<br>3. Widowed<br>4. Never-married /never lived together<br>5. Other (SPECIFY)…………………
**1.5**	Education	1. No formal schooling<br>2. Primary incomplete.<br>3. Primary complete<br>4. Secondary incomplete<br>5. Secondary complete<br>6. College/University incomplete<br>7. College/University complete
**1.6**	Occupation	1. Public servant<br>2. Private formal sector<br>3. Subsistence farmer<br>4. Large scale farmer<br>5. Self employed/small business<br>6. Self employed / large business<br>7. Taking care of the home/children<br>8. Student<br>9. Retired<br>10. Looking for work<br>8. 11. Other______(SPECIFY)
**1.7**	Does your family have a history of diabetes?	☐ 1 Yes ☐2 No
**1.8**	What is the religion of the household head?	1. Catholic<br>2. Protestant/other Christian<br>3. Hindu<br>4. Muslim<br>5. Traditional<br>6. No religion<br>7. Other (specify)
**SECTION 2 Perceived risk and actions in response to COVID-19**		
2.1	Have you heard of COVID-19 or Coronavirus	☐ 1 Yes ☐2 No ☐ Don't know
2.2	Have you been in close contact with someone confirmed to be infected with COVID19	☐ 1 Yes ☐2 No ☐ Don't know
2.3	If yes, where did this contact take place?	1-Healthcare setting<br>2-At home<br>3-Workplace<br>4-Public transport<br>5-Other (Specify)<br>____________________________________
2.4	What did you do after realizing you had been in contact with someone infected with COVID19	1- Did nothing<br>2- Isolation<br>3- Washed hands<br>4- Used sanitizer<br>5- Took treatment<br>6- Others-Specify_______
2.5	Have you noticed any of the following since the start of the pandemic (select all that apply)	1. High temperature- $\geq 38^0$C or subjective fever?<br>2. Persistent dry cough?<br>3. Sore throat or pain when swallowing?<br>4. Breathlessness or a difficulty breathing?

		5. Pain in your body, especially your muscles hurting more than usual?<br>6. Recent changes in your ability to taste or smell things?<br>7. Unusual nausea or vomiting?<br>8. Diarrhea?<br>9. Feeling more tired/sleepy/unable to concentrate/physical weakness than usual?
2.6	Have you ever been told you have COVID-19 by a professional health worker?	☐ 1 Yes ☐2 No
2.7	Have you been tested for COVID- 19? *(SELECT ONLY ONE)*	☐ 1 Yes ☐2 No
2.8	Outcome of the test *(SELECT ONLY ONE)*	☐ Positive ☐ Negative ☐ Don't know
**2.9.**	Have you been vaccinated for COVID-19?	☐1 Yes<br>☐2 No

<table>
<tr><th colspan="3">SECTION 3: IMPACT OF COVID-19 ON HEALTHCARE EXPENDITURE, RESOURCE USE, ACCESS TO HEALTH CARE, SOCIAL SUPPORT, AFFORDABILITY OF MEDICINE</th></tr>
<tr><td>[PRE-COVID] period = before March 2020

INSERT BEFORE MARCH 2020 TO ALL QUESTIONS in SECTIONS 3A, 3B and 3C. Responses should relate to within a 3-month timeframe before March 2020.</td><td colspan="2">Has this changed in [COVID] period = the time you were most affected [if applicable]

If yes, please ask when this happened (month(s))?________________

INSERT NAMED MONTH/PERIOD TO ALL QUESTIONS in SECTIONS 3A, 3B and 3C. Responses should all relate to that named timeframe.</td></tr>
<tr><td colspan="3">Blood sugar test</td></tr>
<tr><td>3.1a Please think about the [PRE–COVID] period. How did you usually test your blood sugar?
(a) At home
(b) Health facility (Hospital/other facility)
(c) Both</td><td>3.1b Has this changed in the [COVID] period?
(a) YES
(b) NO
If the reply is YES, answer 2.1c</td><td>3.1c Please think about the [COVID] period. How do you usually test your blood sugar?
(a) At home
(b) Health facility (Hospital/other facility)
(c) Both</td></tr>
<tr><td>3.2a How often did you test your blood sugar during the [PRE–COVID] period?
(a) More than twice a day
(b)Twice a day
(c)Once a day
(d)Once or twice a week
(e) Less than once a week
(f)Once a month
(g) Once in 3 months</td><td>3.2b Has this changed in the [COVID] period?
(a) YES
(b) NO
If the reply is YES, please answer 2.2c</td><td>3.2c How often do you test your blood sugar during the [COVID] period?
(a) More than twice a day
(b)Twice a day
(c)Once a day
(d)Once or twice a week
(e) Less than once a week
(f)Once a month
(g) Once in 3 months</td></tr>
<tr><td></td><td></td><td>Note: question 3.3c needs to be answered only if the participant reports a decrease in the frequency of blood sugar testing

3.3c If during the [COVID] period you experienced a decrease in the frequency your blood sugar was tested, could you please indicate the main reasons:

(a) testing strips not available/not managed to get all testing strips needed
(b) testing strips too expensive/cannot afford
(c) unable to access the clinic
(d) nurse/doctor not available
(e) other (please explain)
(f) NA</td></tr>
<tr><td>3.4a In the [pre-COVID] period, how much did you usually pay to have your blood sugar tested at the <u>hospital/other facility</u>? (<u>Consider cost/test</u>). Enter zero if not required to pay.
[INSERT AMOUNT IN TZS]
a) At hospital, TZS__________
b) At other facility, TZS______</td><td>3.4b Has this changed in the [COVID] period?
(a)YES
(b)NO

If the reply is YES, please answer 3.4c</td><td>3.4c In the [COVID] period, how much did you usually pay to have your blood sugar tested at the <u>hospital/other facility</u>? (<u>Consider cost/test</u>) Enter zero if not required to pay.
[INSERT AMOUNT IN TZS]
a) At hospital, TZS______
b) At other facility, TZS___</td></tr>
</table>

**3.5a** In the **[pre-COVID]** period, how much did you usually spend to have your blood sugar tested <u>at home</u>? (**Consider cost/test**)<br><br>*[INSERT AMOUNT IN TZS]*	**3.5b** Has this changed in the **[COVID]** period?<br>*(a)YES*<br>*(b)NO*<br><br>**If the reply is YES, please answer 2.5c**	**3.5c** In the **[COVID]** period, how much did you usually spend to have your blood sugar tested <u>at home</u>? (**Consider cost/test**)<br><br>*[INSERT AMOUNT IN TZS]*
**Medications**		
**[PRE-COVID] period = before March 2020**	**Has this changed in [COVID] period = the time you were most affected [if applicable]**	
**3.6a** What type of <u>medication did your doctor prescribe</u> for the treatment and care of diabetes in the **[pre-COVID]** period?<br>(a) Insulin<br>(b) Oral Hypoglycaemic drug (e.g Metformin, Glibenclamide *[country-specific examples*])<br>(c) Insulin + Oral Hypoglycaemic drug<br><br>If the reply is (B) (Hypoglycaemic drug), please insert name of drug below<br>___________________________	**3.6b** Has this changed in the **[COVID]** period?<br>(a) YES<br>(b) NO<br><br>**If reply is YES, please answer 2.6c**	**3.6c** Which medication did <u>your doctor prescribe</u> for the treatment and care of diabetes during the **[COVID]** period?<br>(a) Insulin<br>(b) Oral Hypoglycaemic drug e.g Metformin, Glibenclamide *[country-specific examples*])<br>(c) Insulin + Oral Hypoglycaemic drug<br><br>If the reply is (B) (Hypoglycaemic drug), please insert name of drug below<br>___________________________
<u>**Think about the medication you mentioned in question 3.6a (insulin or hypoglycaemic drug) and reply to the following questions:**</u>		<u>**Think about the medication you mentioned in question 3.6c (insulin or hypoglycaemic drug) and reply to the following questions:**</u>
**3.7a**. <u>Did you use this medication</u> in the [pre-COVID] period?<br>(a)YES<br>(b)NO		**3.7c** <u>Did you use this medication</u> in the [COVID] period?<br>(a)YES<br>(b)NO
**If the reply to 3.7a is YES:**<br><br>**3.8a** Think about the 3 months [pre-COVID] ie between Jan and March 2020**.** Please indicate medication usage:<br><br>(a)Frequency (e.g., once/day; twice/month)<br>(b)Dose (e.g., Mls if insulin or number of tablets)/mg?<br>(c) Duration (e.g. used for only one month; for three months)		**If the reply to 3.7c is YES:**<br><br>**3.8c** Think about the 3 months after **[COVID].** Please indicate:<br><br>(a)Frequency (e.g., one/day; twice/month)<br>(b) Dose (e.g., Mls if insulin or number of tablets)/mg?<br>(c)Duration (e.g., used for only one month; for three months)
**3.9a** How much did you usually spend on insulin/medication in a <u>typical month</u> in the **[pre-COVID]** period?<br>*[INSERT AMOUNT IN TZS]*<br>*a. Insulin monthly cost, TZS__________*<br>*b. Medication monthly cost, TZS______*	**3.9 b**. Has this changed in the [COVID] period?<br>(a) YES<br>(b) NO	**3.9c** How much do you usually spend on insulin/medication in a <u>typical month</u> during the **[COVID]** period?<br>*[INSERT AMOUNT IN TZS]*<br>*a. Insulin monthly cost, TZS__________*<br>*b. Medication monthly cost, TZS______*

<table>
<tr>
<td>3.10a Were you able to obtain all the [insulin/medication] that you needed in the [pre-COVID] period?
a. YES
b. NO

If the reply is NO, please answer 3.11a</td>
<td>3.10 b. Has this changed in the [COVID] period?
(c) YES
(d) NO</td>
<td>3.10c Were you able to obtain all the [insulin/medication] that you needed in the [COVID] period?
a. YES
b. NO

If the reply is NO, please answer 3.11c</td>
</tr>
<tr>
<td>3.11a Please indicate the main reason why you were unable to obtain all the medications/insulin you needed in the [pre-COVID] period
(multiple responses allowed)
(a) too expensive-cannot afford
(b) not available or in stock
(c) poor quality
(d) syringes not in stock
(e) syringes too expensive
(f) don't know how to inject insulin and doctor/nurse not available at the clinic
(g) other reason (please explain)</td>
<td></td>
<td>3.11c Please indicate the main reasons why you are unable to obtain all the medications/insulin during the [COVID] period?
(multiple responses allowed)
(a) too expensive-cannot afford
(b) not available or in stock
(c) poor quality
(d) syringes not in stock
(e) syringes too expensive
(f) don't know how to inject insulin and doctor/nurse not available at the clinic
(g) Afraid to go out due to fear of COVID-19 infection
(h) Lockdown restricted me from going out
(i) Unable to go out for other reason
(j) Limited facility opening hours
(k) other reason (please explain)</td>
</tr>
</table>

SECTION 3B: HOSPITAL ADMISSIONS AND HOSPITAL OUTPATIENT VISITS					
3.12 Please consider the 3 months before COVID. During this period did you ever visit a hospital to get any kind of medical care for yourself, including just seeing a doctor or nurse or just to get medication for diabetes related treatment/complications?<br>(a) *YES (b) NO*<br>**If the reply to Question 2.12 is YES, please reply to questions 3.13, 3.14 and 3.15**<br>**If the reply to Question 2.12 is NO, please skip questions 3.13, 3.14 and 3.15 and go to question 3.16**					
**Consider the [PRE-COVID] period [BEFORE MARCH 2020]. Think about Hospital admissions and hospital (clinic) outpatient visits you had in the 3 months before COVID:**					
**3.13 Hospital admissions** *(the patient has spent more than 24 hours in a bed/stretcher)*	**Indicate number of admissions**	**Number of nights in total (summing up all admissions)**	**Facility Type**<br>(1) Public<br>(2) Private for profit<br>(3) FBO/NGO	**Please indicate reason for admission:**<br>***Diabetes-related tests and medications:***<br>(1) Urine test<br>(2) Blood test<br>(3) finger-prick blood test<br>(4) Blood pressure measurement<br>(5) eye exam<br>(6) test of your feet for feeling<br>(7) taking your weight on a scale<br>(8) measuring your waist with a tape<br>(9) Collect medications<br>(10) Other (please specify)	**Covered by:** (please choose the most used/relevant option)<br>(1) Free treatment<br>(2) Health insurance<br>(3) Own cash<br>(4) Parents support<br>(5) Other family support<br>(6) Had to work for provider<br>(7) Selling an asset<br>(8) Took loan<br>(9) Got assistance<br>(10) Deferred by provider<br>(11) Other (please specify)
**3.14 Hospital admissions** *(the patient has spent less than 24 hours in a bed or stretcher, for example in an observation room, emergency room, or casualty room)*	**Indicate number of admissions**		**Facility Type**<br>(1) Public<br>(2) Private for profit<br>(3) FBO/NGO	**Please indicate reason for admission?:**<br>***Diabetes-related tests and medications:***<br>(1) Urine test<br>(2) Blood test<br>(3) finger-prick blood test<br>(4) Blood pressure measurement<br>(5) eye exam<br>(6) test of your feet for feeling<br>(7) taking your weight on a scale<br>(8) measuring your waist with a tape<br>(9) Collect medications<br>(10) Other (please specify)	**Covered by:** (please choose the most used/relevant option)<br>(1) Free treatment<br>(2) Health insurance<br>(3) Own cash<br>(4) Parents support<br>(5) Other family support<br>(6) Had to work for provider<br>(7) Selling an asset<br>(8) Took loan<br>(9) Got assistance<br>(10) Deferred by provider<br>(11) Other (please specify

**3.15 Outpatients visit**	**Indicate number of visits**		**Facility Type**<br>(1) Public<br>(2) Private for profit<br>(3) FBO/NGO	**Please indicate reason for visit?:**<br>***Diabetes-related tests and medications:***<br>(1) Urine test<br>(2) Blood test<br>(3) finger-prick blood test<br>(4) Blood pressure measurement<br>(5) eye exam<br>(6) test of your feet for feeling<br>(7) taking your weight on a scale<br>(8) measuring your waist with a tape<br>(9) Collect medications<br>(10) Other (please specify)	**Covered by:** (please choose the most used/relevant option)<br>(1) Free treatment<br>(2) Health insurance<br>(3) Own cash<br>(4) Parents support<br>(5) Other family support<br>(6) Had to work for provider<br>(7) Selling an asset<br>(8) Took loan<br>(9) Got assistance<br>(10) Deferred by provider<br>(11) Other (please specify)
3.16 Now please consider the 3 (worst) months during COVID. During this period did you ever visit a hospital to get any kind of medical care for yourself, including just seeing a doctor or nurse or just to get medication for diabetes related treatment/complications?<br>*(a) YES (b) NO*<br>**If the reply to Question 2.16 is YES, please reply to questions 2.17, 2.18, 2.19**<br>**If the reply to Question 2.16 is NO, please skip questions 2.17, 2.18, 2.19 and reply to questions 2.20**					
**Consider the [COVID] period. Think about the Hospital admissions and hospital (clinic) outpatient visits you had in the months during the [COVID period]::**					
**3.17 Hospital admissions**<br>*(the patient has spent more than 24 hours in a bed/stretcher*)	**Indicate number of admissions**	**Number of nights in total (summing up all admissions)**	**Facility Type**<br>(1) Public<br>(2) Private for profit<br>(3) FBO/NGO	**Please indicate reason for admission?:**<br>***Diabetes-related tests and medications:***<br>(1) Urine test<br>(2) Blood test<br>(3) finger-prick blood test<br>(4) Blood pressure measurement<br>(5) eye exam<br>(6) test of your feet for feeling<br>(7) taking your weight on a scale<br>(8) measuring your waist with a tape<br>(9) Collect medications<br>(10) Other (please specify)	**Covered by:** (please choose the most used/relevant option)<br>(1) Free treatment<br>(2) Health insurance<br>(3) Own cash<br>(4) Parents support<br>(5) Other family support<br>(6) Had to work for provider<br>(7) Selling an asset<br>(8) Took loan<br>(9) Got assistance<br>(10) Deferred by provider<br>(11) Other (please specify)
**3.18 Hospital admissions**<br>*(the patient has spent less than*	**Indicate number of admissions**		**Facility Type**<br>(1) Public	**Please indicate reason for admission?:**<br>***Diabetes-related tests and***	**Covered by:** (please choose the most used/relevant option)

24 hours in a bed or stretcher, for example in an observation room, emergency room, or casualty room)			(2) Private for profit<br>(3) FBO/NGO	***medications:***<br>(1) Urine test<br>(2) Blood test<br>(3) finger-prick blood test<br>(4) Blood pressure measurement<br>(5) eye exam<br>(6) test of your feet for feeling<br>(7) taking your weight on a scale<br>(8) measuring your waist with a tape<br>(9) Collect medications<br>(10) Other (please specify)	(1) Free treatment<br>(2) Health insurance<br>(3) Own cash<br>(4) Parents support<br>(5) Other family support<br>(6) Had to work for provider<br>(7) Selling an asset<br>(8) Took loan<br>(9) Got assistance<br>(10) Deferred by provider<br>(11) Other (please specify)
**3.19 Outpatients visit**	**Indicate number of visits**		**Facility Type**<br>(1) Public<br>(2) Private for profit<br>(3) FBO/NGO	**Please indicate reason for visit?:**<br>***Diabetes-related tests and medications:***<br>(1) Urine test<br>(2) Blood test<br>(3) finger-prick blood test<br>(4) Blood pressure measurement<br>(5) eye exam<br>(6) test of your feet for feeling<br>(7) taking your weight on a scale<br>(8) measuring your waist with a tape<br>(9) Collect medications<br>(10) Other (please specify)	**Covered by:** (please choose the most used/relevant option)<br>(1) Free treatment<br>(2) Health insurance<br>(3) Own cash<br>(4) Parents support<br>(5) Other family support<br>(6) Had to work for provider<br>(7) Selling an asset<br>(8) Took loan<br>(9) Got assistance<br>(10) Deferred by provider<br>(11) Other (please specify)

**SECTION 3C: NON-HOSPITAL VISITS**
**3.20** Think about the health care you received at places other than a hospital in the **[pre-COVID]** period [BEFORE **MARCH 2020]**. Have you ever received **in the 3 months** before COVID healthcare from one of the following: specialist doctor or surgeon, primary care doctor, nurse, pharmacist or dispensary, health educator, medical assistant/clinical officer, community health worker?

(a)YES (b)NO
If the reply to question 3.20 is YES, please answer 3.21, 3.22, 3.23, 3.24, 3.25, 3.26, 3.27
If the reply is NO, please go to 3.30

Non hospital admissions and hospital (clinic) outpatient visits you had in the 3 months before COVID:

	**Indicate number of visits**	**Type**<br>1 Public<br>2 Private for profit<br>3 FBO or NGO	**Total fees and charges**	**Please indicate reason:**<br>***Diabetes-specific tests and medications:***<br>(1) Urine test<br>(2) Test on your blood taken by needle<br>(3) finger-prick test for blood sugar<br>(3) Blood pressure measurement<br>(4) eye exam<br>(5) test of your feet for feeling<br>(6) taking your weight on a scale<br>(7) measuring your waist with a tape<br>(8) Collect medications<br>(9) Other (please specify)	**Covered by:** (please choose the most used/relevant option)<br>(1) Free treatment<br>(2) Health insurance<br>(3) Own cash<br>(4) Parents support<br>(5) Other family support<br>(6) Had to work for provider<br>(7) Selling an asset<br>(8) Took loan<br>(9) Got assistance<br>(10) Deferred by provider<br>(11) Other (please specify)
**3.21 Specialist doctor or surgeon**					
**3.22 Primary care doctor**					
**3.23 Nurse**					
**3.24 Pharmacist or dispensary**					
**3.25 Health educator (e.g. diabetes educator)**					
**3.26 Medical assistant/clinical officer**					
**3.27 Community health worker**					

3.30 During the 3 months in the **[PRE–COVID]** period **[BEFORE MARCH 2020**] did you visit a traditional healer's or faith dwelling?(a) YES (b) NO
If the reply is YES, please answer question 3.31
If the reply is NO, please go to question 3.32.

<table>
<tr><td>3.31 Traditional healer/faith dweller/herbalist</td><td>Indicate number of visits</td><td>Total fees and charges</td><td>Please indicate reason:
(1) Treatment
(2) Collect medications
(3) Spiritual healing
(4) Other (please specify)</td><td>Covered by: (please choose the most used/relevant option)
(1) Free treatment
(3) Own cash
(4) Parents support
(5) Other family support
(6) Had to work for provider
(7) Selling an asset (including goat, sheep, Chicken, cows)
(8) Took loan
(9) Got assistance
(10) Deferred by provider
(11) Other (please specify)</td></tr>
<tr><td colspan="5">3.32 Think about the health care you received at places other than a hospital In the [3 worst months of the COVID] period. Have you ever received [during that period] healthcare from one of the following: specialist doctor or surgeon, primary care doctor, nurse, traditional healer, pharmacist or dispensary, health educator, medical assistant/clinical officer, community health worker?
(a) YES (b) NO
If the reply to question 3.32 is YES, please answer questions 3.33, 3.34, 3.35, 3.36, 3.37, 3.38, 3.39
If the reply to question 3.32 is NO, go to 3.40</td></tr>
<tr><td colspan="5">Think about the Non-Hospital visits you had in <u>the months during the [COVID period]</u>:</td></tr>
</table>

	Indicate number of visits	Type<br>1 Public<br>2 Private for profit<br>3 FBO or NGO	Total fees and charges	**Please indicate reason:**<br>***Diabetes-specific tests and medications:***<br>(1) Urine test<br>(2) Test on your blood taken by needle<br>finger-sick blood test<br>(3) Blood pressure measurement<br>(4) eye exam<br>(5) test of your feet for feeling<br>(6) taking your weight on a scale<br>(7) measuring your waist with a tape<br>(8) Collect medications<br>(9) Other (please specify)	**Covered by:** (please choose the most used/relevant option)<br>(1) Free treatment<br>(2) Health insurance<br>(3) Own cash<br>(4) Parents support<br>(5) Other family support<br>(6) Had to work for provider<br>(7) Selling an asset (including goat, sheep, chicken, cows)<br>(8) Took loan<br>(9) Got assistance<br>(10) Deferred by provider<br>(11) Other (please specify)
**3.33 Specialist doctor or surgeon**					
**3.34 Primary care doctor**					
**3.35 Nurse**					
**3.36 Pharmacist or dispensary**					
**3.37 Health educator (e.g. diabetes educator)**					
**3.38 Medical assistant/clinical officer**					
**3.39 Community health worker**					

3.40 In the months during the [COVID period] did you/[person] visit a traditional healer's or faith dwelling?
(a) YES (b) NO

If the reply to question 3.40 is YES, please reply question 3.41
If the reply to question 3.40 is NO, please reply question 3.42

**3.41 Traditional healer/faith dweller/herbalist**	**Indicate number of visits**	**Total fees and charges**	**Please indicate reason:**<br>(1) Treatment<br>(2) Collect medications<br>(3) Spiritual healing<br>(4) Other (please specify)	**Covered by:** (please choose the most used/relevant option)<br>(1) Free treatment<br>(3) Own cash<br>(4) Parents support<br>(5) Other family support<br>(6) Had to work for provider<br>(7) Selling an asset (including goat, sheep, chicken, cows)<br>(8) Took loan<br>(9) Got assistance<br>(10) Deferred by provider<br>(11) Other (please specify)

SECTION 3D: DIABETES SELF-CARE

3.42 Since COVID-19 cases were identified in [Country], which of the following have affected your diabetes self-care? (Tick all that apply)

(a) Difficulty in securing appointments with a doctor
(b) Hesitation leaving home due to risk of COVID-19 infection
(c) Difficulty in buying infection control supplies such as sanitizer, masks
(d) Difficulty seeking community and social services
(e) Transport cost too high
(f) Lack of money to buy medications
(g) Lack of covid 19 vaccination?
(h) Other (Specify)

SECTION 3E: ACCESS TO HEALTH CARE

We are interested to know whether the COVID pandemic has affected the accessibility to the main healthcare facilities you used to manage your T2D. Please think about the main healthcare facility/healthcare provider you used to get T2D -related care in the period [before COVID].

3.43 What is the main healthcare facility you used **[before COVID JANUARY – MARCH 2020]** to get TD2-related care?

**Healthcare facility by ownership**	(1) Public<br>(2) Private for profit<br>**(3)** Charity/NGO/ faith-based
**Healthcare facility by level of care**	(1) Hospital<br>(2) Health centre<br>(3) Dispensary<br>(4) Pharmacy
**Other** (please indicate)	

3.44 Were you usually successful in seeing this healthcare provider at the health facility you visited? **[before COVID MAR 2020]**?

a) Yes
b) No, tried but could not be seen

If reply is YES, please answer question 2.45
If reply is NO, please answer question 2.46

3.45 Did you usually have a phone consultation or in person consultation **[before COVID MAR 2020]**?

(a) phone consultation
(b) In-person
(c) Other (e.g., WhatsApp)

3.46 If you were unable to see a healthcare provider **[before COVID MAR 2020]**, please tell us what are the reasons why you were not able to see a healthcare provider at the facility you visited? [tick all that apply]

(a) Unable to go out
(b) Unable to afford
(c) Clinic closed
(d) Other reason (specify) __________

Now think about the [COVID] period ie the time you were most affected

3.47 What is the main healthcare facility you used **[during COVID]** to get TD2-related care?

**Healthcare facility by ownership**	(1) Public

	(2) Private for profit<br>**(3)** Charity/NGO/ faith-based
**Healthcare facility by level of care**	(1) Hospital<br>(2) Health centre<br>(3) Dispensary<br>(4) Pharmacy
**Other** (please indicate)	

3.48 Were you successful in seeing this healthcare provider during the **[COVID]** period?

a) Yes
b) No, tried but could not be seen

If reply is YES, please answer question 2.49
If reply is NO, please answer question 2.50

3.49 Did you usually have a phone consultation or in-person consultation during the **[COVID]** period?

(a) phone consultation
(b) In-person
(c) Other (e.g., WhatsApp)

3.50 If you were unable to see a healthcare provider during the **[COVID]** period, please tell us what are the reasons why you were not able to see a healthcare provider at the facility you visited? [tick all that apply]

(a) Afraid to go out due to fear of COVID-19 infection
(b) Lockdown restricted me from going out
(c) Unable to go out for other reason
(d) Unable to afford
(e) Clinic closed
(f) Other reason (specify) __________

If reply to question 3.48 is NO

3.51 Considering that during the [COVID] period you were not successful in seeing your usual healthcare provider, did you seek healthcare in another facility?

YES
NO

If the reply is YES, please answer 3.52a, 3.53a, 3.54a, 3.52b, 3.53b, 3.54b.
If the reply is NO, please reply answer only 3.52b, 3.53b, 3.54b.

**Consider the main healthcare facility used in the PRE–COVID period:**	**Consider the main healthcare facility used in the COVID period:**
3.52a How long did the journey to and from the healthcare facility take?<br>*INDICATE DURATION IN HOURS AND/OR MINUTES, CONSIDERING ROUND TRIP*	3.52b How long did the journey to and from the healthcare facility take?<br>*INDICATE DURATION IN HOURS AND/OR MINUTES, CONSIDERING ROUND TRIP*

**3.53a** How did you usually travel to the hospital/health facility?<br><br>(1) Walk<br>(2) Bicycle<br>(3) Motorcycle<br>(4) Tricycle<br>(5) Private car<br>(6) Public taxi/bus<br>(7) Other (specify)	**3.53b** How did you usually travel to the hospital/health facility?<br><br>(1) Walk<br>(2) Bicycle<br>(3) Motorcycle<br>(4) Tricycle<br>(5) Private car<br>(6) Public taxi/bus<br>(7) Other (specify)
**3.54a** Did you pay for the journey to the health facility? If yes, how much in total did you pay for yourself for a round trip?	**3.54b** Did you pay for the journey to the health facility? If yes, how much in total did you pay for yourself for a round trip?

SECTION 4: IMPACT OF COVID-19 ON INCOME, IMPOVERISHMENT, AND AVAILABILITY OF FOOD

4.1 Are you covered with any health insurance?
(a)YES
(b)NO

4.2 If yes, which type of health insurance?
(a)NHIF
(b)ICHF
(c) NSSF-SHIB
(d) Private
(e) Other, specify____________

4.3 When did you join the health insurance scheme?
(a) After being diagnosed with T2D
(b) Before being diagnosed with T2D
(c) Before COVID and before being diagnosed with T2D
(d) After COVID-19

4.4 Please indicate your average income **[PRE–COVID]** [Amount in TZS]
_____________per month [net income/salary for formal employees]
Or ____________ per week
Or _____________per day.

4.5 How much per month did you spend on average [PRE-COVID] on general healthcare. Please include medications, outpatients, inpatient, out of pocket costs including transport.

4.6 How much per month did you spend on average [PRE–COVID] to access to healthcare for T2D?

4.7 Have COVID-19 laws/regulations/rules affected the ability of you or your household to earn money at any point? *(SELECT ONLY ONE)*
(a) Yes;
(b) No;
(c) Don't Know

4.8 Which of the following statements are true regarding the ability of you or your household to earn money <u>during the COVID period</u>
Select all that apply,
(a) Someone in the household has lost a job;
(b) Someone in the household is on unpaid leave

(c) Others (Specify)

4.9 Please indicate your average income in the **[COVID] period.** [Amount in TZS/Kshs]
____________/per month [net income/salary for formal employees]
Or ____________ per week
Or _____________per day.

4.10 How much per month you do spent [during COVID] to access healthcare for T2D? On general healthcare resource use?

We might want to phrase it as ‘any changes’ in health care resource use? (& associated time and productivity impacts) Then specify what at the key changes/impacts/costs/time.

4.11 Here is a picture of a ladder. Please think of this ladder as representing the socioeconomic standing of people in your community. At the top of the ladder are those who are best off; they have the most money, the most education, the most respected jobs. At the bottom are people who have the least money, least education, and least jobs.

a) Where would you place yourself on this ladder **[PRE–COVID]**? ___________

b) Where would you place yourself on this ladder in the **[COVID]** period? _____________

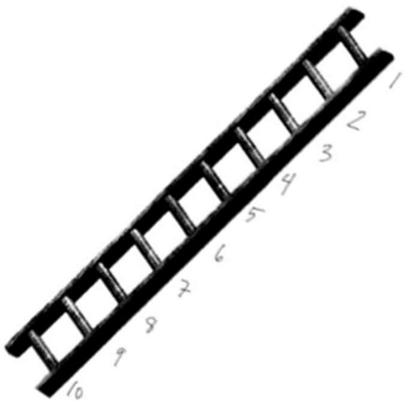

4.12 Is your family capable to get the standard three meals of food they need per day? *(SELECT ONLY ONE)*
(a) Yes;
(b) No;
(c) Don't Know

4.13 What are your coping mechanisms in the case of financial hardship?
Select all that apply.
(a) Not pay bills that are due (e.g., school fees, rent etc.);
(b) Take out a loan;
(c) Sell some assets/ animals/
(d) Skip meals;
(e) Others

SECTION 5: IMPACT OF COVID ON PRODUCTIVITY

5.1 Think about the **[PRE-COVID]** period. How many days in a week on average did you miss your usual activities (work, work at home or school) because of health reasons related to diabetes?

[INSERT NUMBER OF DAYS]

5.2 During the **[COVID period]** has the average number of days you missed your usual activities changed?

(a)YES, increased
(b)YES, decreased
(c)NO, stayed the same

If the reply to Question 4.2 is YES, please reply to Question 4.3

5.3 Think about the **[COVID]** period. How many days in a week on average do you miss your usual activities (work, work at home or school) because of health reasons related to diabetes?

[INSERT NUMBER OF DAYS]

SECTION 6: IMPACT OF COVID ON FORMAL AND INFORMAL CARE

[PRE-COVID] period	**[COVID] period**	**[COVID] period**
Formal Care		
**6.1a** In the [PRE-COVID] period, did you or your family hire someone to take care of you because of your ill health?<br>*(a)YES*<br>*(b)NO*	**6.1b** Has this changed in the [COVID] period?<br>*(a)YES*<br>*(b)NO*	
**6.2a** If the reply to Q1a is YES, Considering the [PRE-COVID] period, how much did you pay this person each month?<br>*[INSERT AMOUNT IN NATIONAL CURRENCY]*	**6.2b** Has this amount changed in the [COVID] period?<br>*(a)YES*<br>*(b)NO*<br><br>***If the reply is YES, please reply to 6.2c***	**6.2c** Considering the [COVID] period, how much do you pay this person each month?<br>*[INSERT AMOUNT IN NATIONAL CURRENCY]*
Informal Care		
**6.3a** In the [PRE-COVID] period, does someone from your family who is not paid usually take care of you because of your ill health?<br>(a)YES<br>(b)NO	**6.3b** Has this changed in the [COVID] period?<br>(a)YES<br>(b)NO	
**6.4a** Considering the [PRE-COVID] period, if the reply to Q3a is YES, how many hours/days did this person spend taking care of you?<br>*[INSERT NO. HOURS]*	**6.4b** Has the number of hours/days this person spends taking care of you changed [pOST-COVID]?<br>YES<br>NO<br>***If the reply is YES, please reply to Q4c***	**6.4c** Considering the [COVID] period, how many hours/days does this person spend taking care of you?<br>*[INSERT NO. HOURS]*
