# Supplementary material for: Economic impact of COVID-19 on patients with type 2 diabetes in Kenya and Tanzania: a costing analysis
Source: BMJ Public Health. 2024 Aug 24;2(2):e000383. doi: 10.1136/bmjph-2023-000383 (PMC11816087; doi:10.1136/bmjph-2023-000383)
Supplement: online supplemental file 2 [file bmjph-2-2-s002.pdf]

APPENDICES

APPENDIX A: Full descriptive statistics of direct costs (total, healthcare, and transport) and indirect costs

		Pre-COVID-19					During COVID-19					P-value
**Country**	**Variable**	**Mean**	**Std. Dev.**	**Median**	**Min**	**Max**	**Mean**	**Std. Dev.**	**Median**	**Min**	**Max**	
**TANZANIA (in I$)**	Total direct cost	19.17	25.92	4.77	0	203.01	19.33	27.16	5.5	0	221.2	0.927
	Healthcare cost	15.66	25.24	0	0	202.11	15.45	26.27	0	0	206.6	0.895
	Transport cost	3.51	5.11	2.25	0	44.91	3.88	6.09	2.25	0	67.37	0.300
	Cost of testing	3.48	8.35	0	0	101.06	4.27	8.94	0	0	101.06	0.146
	Cost of medications	14.83	22.76	0	0	168.43	14.89	26.86	0	0	359.31	0.973
	Hospitalisation cost	17.62	71.8	0	0	846.24	19.26	55.94	0	0	564.16	0.689
	Outpatient cost	33.6	28.04	45.48	0	229.92	47.89	76.04	45.48	0	459.84	0
	Informal care costs	1886.28	1468.12	1438.94	479.65	5755.78	1702.58	1500.25	1438.94	0	5755.78	0.2996
	Productivity loss costs	930.29	1640.87	0	0	7902.44	1155.13	1783.27	0	0	7902.44	0.0383
**KENYA (in I$)**	Total direct cost	116.88	247.07	68.51	0	5023.98	120.29	154.13	73.08	0	1370.18	0.794
	Healthcare cost	104.58	226.13	54.81	0	4567.25	106.79	143.81	68.51	0	1256	0.853
	Transport cost	12.31	31.76	4.57	0	456.73	13.50053	27.74	4.57	0	228.36	0.527
	Cost of testing	20.11	46.58	0	0	342.54	23.85	49.62	0	0	228.32	0.493
	Cost of medications	85.16	110.36	23.98	0	822.11	76.37	223.11	22.83	0	4,567.16	0.430
	Hospitalisation cost	0.84	5.6	0	0	94.34	478.67	10700.49	0	0	239,780.77	0.000
	Outpatient cost	59.74	194.04	3.31	0	2,283.58	66.9	261.82	2.97	0	3,425.44	0.000
	Informal care costs	12967.7	2455.68	0	0	26083.20	13009	1364.47	6416.8	2173.60	26083.2	0.7424
	Productivity loss costs	3324.45	4954.48	13041.6	0	22458.8	6471.88	7172.446	13041.6	0	22458.8	0.000

Notes: All costs expressed in I$

APPENDIX B: Full Regression models for Kenya and Tanzania

APPENDIX B1: Full regression outputs for a two-part model including the probability of incurring costs (odds ratio for logit) and GLM coefficient for each direct cost category in Tanzania.

	**Direct total cost**		**Healthcare cost**		**Transport cost**		**Cost of testing**		**Cost of medication**		**Hospitalization cost**		**Outpatient cost**	
	**LOGIT (OR)**	**GLM (Coef.)**	**LOGIT (OR)**	**GLM (Coef.)**	**LOGIT (OR)**	**GLM (Coef.)**	**LOGIT (OR)**	**GLM (Coef.)**	**LOGIT (OR)**	**GLM (Coef.)**	**LOGIT (OR)**	**GLM (Coef.)**	**LOGIT (OR)**	**GLM (Coef.)**
Time (during COVID-19)	0.873**	-0.004	0.736***	0.055*	0.903***	0.106***	0.981	0.221***	0.786**	0.045	1.487**	-0.182*	1.023	0.213***
Rural (ref: urban)	0.730	-0.431***	0.684	-0.529***	0.717	-0.261**	0.202***	0.666***	0.620	-0.363**	1.945***	0.124	6.341***	0.640***
Male (ref: female)	0.694	0.104	1.947**	0.041	0.606*	-0.288**	1.620	-0.105	1.093	-0.037	0.872	0.052	1.192	-0.012
Married (ref: not married)	1.094	0.286**	1.065	0.175	0.980	0.347***	0.659	0.050	1.204	0.063	1.471*	-0.077	1.031	0.085
40-49 years (ref: <40 years)	6.072**	0.114	2.633*	0.141	4.787***	-0.599**	0.812	-0.132	1.234	-0.078	0.532	0.185	0.986	-0.178*
50-59 years	4.065***	-0.300*	1.884	-0.172	3.208**	-0.610**	0.565	-0.115	0.724	-0.201	0.495**	0.092	1.163	-0.059
60-69 years	3.167**	-0.361*	0.940	-0.062	3.513***	-0.572**	0.251**	-0.203	0.600	-0.079	0.416**	-0.020	1.103	-0.064
> 70 years	1.210	-0.637**	1.038	-0.367	1.324	-0.816**	0.068**	1.768**	0.163*	0.157	0.411	0.064	1.079	0.203
Primary (ref: no education)	1.090	0.171	1.008	0.139	1.211	0.371**	0.720	0.107	0.592	0.157	0.577	-0.141	2.033*	-0.130
Secondary	1.113	-0.159	0.952	-0.078	1.531	0.331*	0.481	0.221	0.342**	0.179	0.541	-0.255	1.657	-0.230**
College/University	1.044	-0.015	0.776	0.393	1.166	0.479**	0.314	-0.274	0.323	0.602**	0.437	0.084	1.479	-0.225*

Protestants (ref: Catholic)	0.700	-0.097	0.916	-0.114	0.904	0.039	0.755	0.510**	0.797	-0.002	0.796	0.102	1.982**	0.010
Muslims	1.179	-0.359***	0.680	-0.153	1.359	-0.245*	0.436**	0.231	0.819	0.012	0.746	-0.038	2.849***	-0.051
Farmer (ref: formal workers)	0.696	0.556**	0.723	0.498	0.609	0.669***	1.407	-0.125	0.687	0.093	0.521	0.532***	0.522	0.195*
Self-employed business	0.883	0.337	1.537	0.120	0.594	0.002	1.439	0.019	1.147	-0.221	0.690	0.472***	0.804	0.068
Retired	0.601	0.308	1.043	-0.371	0.415	0.230	1.850	-0.074	0.622	-0.652**	0.947	0.887***	0.897	0.117
Unemployed	0.213**	0.405	0.752	0.254	0.175***	0.131	2.490	-0.075	0.758	-0.035	0.693	0.357*	0.822	0.173
Insured (ref: uninsured)	0.484**	-1.220***	0.053***	-0.353**	0.752	-0.029	0.036***	-0.160	0.035***	-0.077	1.385	-0.343**	1.174	0.082
SES lower (ref: higher SES)	0.606	0.371***	1.670*	0.238	0.585*	0.036	0.907	-0.234	1.531	0.202	1.532*	-0.079	0.963	-0.109
Family history of T2D (ref: no history)	0.603*	-0.035	1.035	-0.063	0.544**	-0.060	1.028	0.067	1.146	-0.004	0.940	0.429***	1.296	0.043
Having T2D <6years (ref: >6 years)	1.360	-0.055	1.351	0.047	0.999	-0.102	1.229	0.024	0.822	-0.085	0.945	0.007	1.322	0.004
# Of comorbidities	0.835	-0.006	1.150	-0.037	0.853	0.048	0.990	-0.014	0.941	0.091*	1.195	0.119***	0.695***	-0.023
Constant	13.008	3.269	2.234	3.297	9.792	1.510	51.050	1.890	16.905	3.408	0.244	4.466	0.482	3.646

Notes: *, p-value<0.05, **, p-value ≤ 0.01, ***, p-value≤ 0.001

APPENDIX B2: Full regression outputs for each direct cost category in Kenya showing GLM coefficient

	Total Direct Costs	Healthcare costs	Transport costs	Testing Costs	Medication Costs	Hospitalization Costs	Outpatient Visit costs
**VARIABLES**	**glm(Coeff)**	**glm (Coeff)**	**glm(Coeff)**	**glm(Coeff)**	**glm(Coeff)**	**glm(Coeff)**	**glm(Coeff)**
Time (=1 during COVID)	0.050	0.036	0.131	0.030	-0.224*	-0.880**	0.127
Male (ref: female)	-0.170	-0.197	-0.027	-0.067	-0.194	0.013	-0.067
Rural (ref: urban)	-0.097	-0.063	-0.371**	0.060	0.659***	-1.509**	0.223
Primary Education (ref: no education)	-0.594**	-0.650*	-0.035*	-0.001	-0.402	0.828	-0.983***
Secondary Education	-0.250	-0.281	0.220	0.129	-0.228	1.339	-0.934
College/University Education	-0.141	-0.154	0.108		0.064	-0.707	-0.657
Vaccinated	-0.080	-0.090	-0.098	-0.117	0.030	-0.975**	-0.428
40-49 years (ref: <40 years)	0.124	0.275	-0.808**	0.243*	0.491*	0.472	0.378
50-59 years	0.145	0.281	-0.490*	0.091	0.620**	0.304	0.558
60-69 years	0.347*	0.487**	-0.304	0.138	0.560**	-0.202	0.598*
> 70 years	0.579***	0.758***	-0.250	0.087	0.872***	0.184	0.942***
Less than 6years living with T2D (ref: >6 years)	0.017	0.010	0.059	-0.155*	0.158	-0.333	-0.147
Family History of Diabetes (ref: no history)	-0.134	-0.097	-0.429**	-0.106	0.147	-0.099	0.346*
Protestants (ref: Catholic)	0.081	0.080	0.095	0.082	-0.196	1.274**	0.067
Muslims	0.254	0.352	-0.502	0.140	0.807	0.279	0.136
Lower SES (ref: higher SES)	0.005	-0.043	0.565*	-0.022	-0.574***	2.144***	0.661*
Number of comorbidities	-0.015	-0.018	-0.004	0.005	0.006	-0.265***	-0.007
Married (ref: not married)	0.077	0.074	0.036	0.007	0.030	-0.265	-0.180
Farmers (Small Scale and large Scale) (ref: formal workers)	-0.173	-0.198	0.039	-0.086	-0.210	3.230	-0.817
Self Employed(Small and Large Business	-0.074	-0.087	0.047	-0.014	-0.039	-0.080	-0.598
Retired	-0.073	-0.113	0.129	-0.112	-0.349	1.235	-0.694
Unemployed	0.074	0.042	0.120	0.027	0.122	2.032	-0.460
Not insured (ref: insured))	-0.167	-0.173	-0.126	-0.074	-0.184	-2.814	-0.076
Constant	5.009	4.861	2.504	1.177	4.465	-2.042	4.478

Notes: *, p-value<0.05, **, p-value ≤ 0.01, ***, p-value≤ 0.001

APPENDIX C: Effect of COVID-19 on the likelihood of incurring in/direct costs (probability/ odds-ratio) in Tanzania - descriptives

Dependent variable	Healthcare cost	Transport cost	Total direct cost	Testing cost	Medication cost	Hospitalisation cost	Outpatient cost
	(1)	(2)	(3)	(4)	(5)	(6)	(7)
COVID-19 period							
Odds Ratio	0.74***	0.90***	0.87**	0.98	0.79**	1.49**	1.02
(SE)	(0.066)	(0.034)	(0.051)	(0.082)	(0.081)	(0.275)	(0.099)
Prob. of paying (pre-COVID-19)	46.4%	82.2%	85.2%	45.6%	43.4%	11.8%	70.4%
Pseudo R-squared	0.365	0.084	0.114	0.499	0.467	0.046	0.128
No. of observations	1000	1000	1000	1000	1000	1000	1000

Notes: Robust standard errors (SE) in parentheses; The reference period is pre-COVID-19 period (before March 2020); Adjusted covariates include location, gender, marital status, age, education level, religion, occupation, insurance status, household socioeconomic status, family history of T2D, time living with T2D, and number of comorbidities; * p<0.10, ** p<0.05, *** p<0.01. The conversion rate to USD ($) is the average exchange rate in 2022 for Kenya and Tanzania (Kshs. 113.9=1US $, TZshs. 2325.94=1 US $)

APPENDIX D: **Full regression outputs including the probability of incurring costs (logit) for each indirect cost category in Tanzania and Kenya**

APPENDIX D1: Effect of COVID-19 on Productivity costs in Tanzania

	**Productivity costs, Tanzania Two-part model**	
	(1)	(2)
Variables	Logit	Glm
**Time (=1 during COVID)**	**0.204*** **	**0.122*** **
	**(0.067)**	**(0.041)**
Rural (ref: urban)	0.559**	-0.568***
	(0.226)	(0.104)
Male (ref: female)	0.036	0.113
	(0.214)	(0.094)
Married (ref: not married)	-0.343*	0.070
	(0.197)	(0.080)
40-49 years (ref: <40 years)	-0.388	0.116
	(0.415)	(0.148)
50-59 years	-0.498	-0.126
	(0.367)	(0.141)
60-69 years	-0.379	-0.176
	(0.380)	(0.142)
70+ years	-1.060*	0.214
	(0.547)	(0.206)
not insured (ref: insured)	-0.297	-0.0737
	(0.214)	(0.094)
Primary education (ref: no education)	0.464	0.068
	(0.331)	(0.127)
Secondary education	0.595	-0.600***
	(0.589)	(0.164)
University education	-0.183	-0.067
	(0.372)	(0.131)
Religion catholic (ref: protestant)	0.348	-0.118
	(0.256)	(0.103)
Religion Muslim	0.410*	-0.166*
	(0.249)	(0.093)
Farmer (ref: formal workers)	0.088	-0.079
	(0.390)	(0.151)
Self-employed business	-0.130	0.074
	(0.377)	(0.144)
Retired	-0.044	-0.095
	(0.426)	(0.178)
Unemployed/looking for job	-0.118	-0.047
	(0.423)	(0.180)
SES lower (ref: higher SES)	-0.275	-0.139*
	(0.204)	(0.084)
Family history of T2D (ref: no history)	-0.029	0.104
	(0.186)	(0.070)
Living with T2D (<6years) (ref: >6 years)	-0.076	0.028
	(0.198)	(0.077)
Number of comorbidities	0.146	-0.016
	(0.106)	(0.034)
Constant	-0.338	8.248***
	(0.547)	(0.221)

Observations	1,000	1,000

	Predicted Productivity costs, marginal effects
**Pre-COVID 19**	917.928
**During-COVID 19**	1173.071

APPENDIX D2: Effect of COVID-19 on Productivity Costs in Kenya

	Productivity costs, Kenya Two-part model	
	(1)	(2)
Variables	Logit	Glm
**Time (=1 during COVID)**	0.567***	0.398***
	(0.072)	(0.045)
Rural (ref: urban)	0.234	-0.037
	(0.245)	(0.069)
Male (ref: female)	-0.181	-0.128**
	(0.234)	(0.0621)
Married (ref: not married)	-0.271	0.005
	(0.215)	(0.067)
40-49 year (ref: <40 year)	0.091	-0.118
	(0.413)	(0.108)
50-59 years	-0.729*	-0.136
	(0.392)	(0.103)
60-69 years	-0.568	-0.208**
	(0.385)	(0.101)
70+ years	-0.533	-0.070
	(0.440)	(0.115)
Primary education (ref: no education)	0.119	0.171
	(0.572)	(0.290)
Secondary education	-0.316	0.135
	(0.576)	(0.292)
University education	-0.994	0.250
	(0.639)	(0.304)
Religion catholic (ref: protestant)	0.452*	0.085
	(0.244)	(0.065)
Religion Muslim	1.232**	0.244**
	(0.568)	(0.115)
Farmer (ref: formal workers)	0.397	0.126
	(0.527)	(0.136)
Self-employed business	-0.257	-0.024
	(0.498)	(0.128)
Homemaker	-0.158	0.007
	(0.762)	(0.162)
Retired	-0.073	0.011
	(0.547)	(0.162)
Unemployed/looking for job	-0.577	0.040

	(0.516)	(0.134)
not insured (ref: insured)	-0.025	0.020
	(0.209)	(0.059)
SES lower (ref: higher SES)	0.742**	0.041
	(0.302)	(0.093)
Family history of T2D (ref: no history)	0.379**	-0.004
	(0.189)	(0.056)
Number of comorbidities	0.012	0.008
	(0.026)	(0.008)
Constant	-0.762	8.828***
	(0.939)	(0.336)
Observations	936	936

**Predicted Costs, marginal effects**	
	**Productivity costs**
**Pre COVID-19**	3347.766
**During COVID-19**	6433.638

Notes: *, p-value<0.05, **, p-value ≤ 0.01, ***, p-value≤ 0.001

References

1. Organization WH. The top 10 causes of death 2020. Accessed October 17, 2022. https://www.who.int/news-room/fact-sheets/detail/the-top-10-causes-of-death.

2. Federation. ID. *IDF Diabetes Atlas 10th Edition*.; 2021.

3. Organization WH. Diabetes. Published 2021. https://www.who.int/news-room/facts-in-pictures/detail/diabetes#:~:text=Prevalence is increasing worldwide%2C particularly,widespread lack of physical activity.

4. Liu J, Bai R, Chai Z, Cooper ME, Zimmet PZ, Zhang L. Low-and middle-income countries demonstrate rapid growth of type 2 diabetes: An analysis based on Global Burden of Disease 1990–2019 data. *Diabetologia*. 2022;65(8):1339-1352.

5. Mutyambizi C, Pavlova M, Chola L, Hongoro C, Groot W. Cost of diabetes mellitus in Africa: a systematic review of existing literature. *Global Health*. 2018;14(1):3. doi:10.1186/s12992-017-0318-5

6. Adamjee E, de Dieu Harerimana J. Estimating the Economic Burden of Diabetes Mellitus in Kenya: a Cost of Illness Study. Published online 2022.

7. Beran D. The impact of health systems on diabetes care in low and lower middle income countries. *Curr Diab Rep*. 2015;15(4):20.

8. Bhojani U, Mishra A, Amruthavalli S, et al. Constraints faced by urban poor in managing diabetes care: patients' perspectives from South India. *Glob Health Action*. 2013;6(1):22258.

9. Esterson YB, Carey M, Piette JD, Thomas N, Hawkins M. A systematic review of innovative diabetes care models in low-and middle-income countries (LMICs). *J Health Care Poor Underserved*. 2014;25(1):72-93.

10. Organization WH. The impact of the COVID-19 pandemic on noncommunicable disease resources and services: results of a rapid assessment. Published online 2020.

11. MOHCDGEC. National Health Accounts (NHA) for financial years 2013/14, 2014/15 and 2015/16. Published online 2019.

12. Maina T, Chen A, Perales N. *October 2014 HEALTHCARE FINANCING OPTIONS FOR KENYA*.; 2014.

13. Mirsky JB, Horn DM. Chronic disease management in the COVID-19 era. *Am J Manag Care*. 2020;26(8):329-330.

14. Fadini GP, Morieri ML, Boscari F, et al. Newly-diagnosed diabetes and admission hyperglycemia predict COVID-19 severity by aggravating respiratory deterioration. *Diabetes Res Clin Pract*. 2020;168:108374.

15. Gupta R, Ghosh A, Singh AK, Misra A. Clinical considerations for patients with diabetes in times of COVID-19 epidemic. *Diabetes Metab Syndr*. 2020;14(3):211.

16. Fadini GP, Morieri ML, Longato E, Avogaro dan A. Prevalence and impact of diabetes among people infected with SARS-CoV-2. *J Endocrinol Invest*. 2020;43(6):867-869.

17. Hussain A, Bhowmik B, do Vale Moreira NC. COVID-19 and diabetes: Knowledge in progress. *Diabetes Res Clin Pract*. 2020;162:108142.

18. Schofield J, Leelarathna L, Thabit H. COVID-19: Impact of and on Diabetes. *Diabetes Ther Res Treat Educ diabetes Relat Disord*. 2020;11(7):1429-1435. doi:10.1007/s13300-020-00847-5

19. Tabe-Ojong MPJ, Gebrekidan BH, Nshakira-Rukundo E, Börner J, Heckelei T. COVID-19 in

rural Africa: Food access disruptions, food insecurity and coping strategies in Kenya, Namibia, and Tanzania. *Agric Econ*. Published online 2022.

20. Rodela TT, Tasnim S, Mazumder H, Faizah F, Sultana A, Hossain MM. Economic impacts of coronavirus disease (COVID-19) in developing countries. Published online 2020.

21. Fatoye F, Gebrye T, Arije O, Fatoye CT, Onigbinde O, Mbada CE. Economic Impact of COVID-19 lockdown on households. *Pan Afr Med J*. 2021;40(1).

22. Wangari EN, Gichuki P, Abuor AA, et al. Kenya's response to the COVID-19 pandemic: a balance between minimising morbidity and adverse economic impact. *AAS Open Res*. 2021;4.

23. Mfinanga SG, Mnyambwa NP, Minja DT, et al. Tanzania's position on the COVID-19 pandemic. *Lancet*. 2021;397(10284):1542-1543.

24. Mkupete MJ, Donath LT, Mugizi FMP. Household Resilience to Food and Nutrition Insecurity during COVID-19 in Tanzania. *GeoJournal*. Published online 2022:1-15.

25. Organization WH. WHO Health Emergency.

26. Cochran WG. *Sampling Techniques*. john wiley & sons; 1977.

27. Nath A, Sudarshan KL, Rajput GK, Mathew S, Chandrika KRR, Mathur P. A rapid assessment of the impact of coronavirus disease (COVID-19) pandemic on health care & service delivery for noncommunicable diseases in India. *Diabetes Metab Syndr Clin Res Rev*. 2022;16(10):102607.

28. URT. *National Health Insurance Fund (NHIF) Unit Cost for Mainland Tanzania and Zanzibar, 2017/18.*; 2018.

29. Organization WH. *Prevention and Control of Noncommunicable Diseases: Guidelines for Primary Health Care in Low Resource Settings*. World Health Organization; 2012.

30. Index of Labour. Accessed October 9, 2022. https://www.nbs.go.tz/nbs/takwimu/labour/

31. World bank. World Development indicators.

32. Belotti F, Deb P, Manning WG, Norton EC. twopm: Two-part models. *Stata J*. 2015;15(1):3-20.

33. Quaife M, Van Zandvoort K, Gimma A, et al. The impact of COVID-19 control measures on social contacts and transmission in Kenyan informal settlements. *BMC Med*. 2020;18:1-11.

34. Gathogo J. COVID-19 containment measures and 'prophecies' in Kenya. In: *Religion and the Covid-19 Pandemic in Southern Africa*. Routledge; 2022:126-140.

35. CHATHAM HOUSE. Tanzania Evades COVID-19 Lockdown, but Restrictions Persist.https://www.chathamhouse.org/2020/05/tanzania-evades-covid-19-lockdown-restrictions-persist. Published 2020.

36. Sanya RE, Karugu CH, Binyaruka P, et al. Impact of the COVID-19 pandemic on type 2 diabetes care and factors associated with care disruption in Kenya and Tanzania. *Glob Health Action*. 2024;17(1). doi:10.1080/16549716.2024.2345970

37. Akter F, Haque M, Akter S, et al. Assessing the management of patients with type 2 diabetes in Bangladesh during pre-and post-COVID-19 era and the implications: A pilot study. *J Appl Pharm Sci*. 2022;12(5):88-97.

38. Opanga S, Njeri LW, Kimonge D, Godman B, Oluka M. Assessing utilisation and expenditure on long-acting insulin analogues in Kenya; findings and implications for the future. *Sch Acad J Pharm*. 2021;10(4):63-70.

39. Singh K, Kondal D, Mohan S, et al. Health, psychosocial, and economic impacts of the COVID-19 pandemic on people with chronic conditions in India: a mixed methods study. *BMC Public Health*. 2021;21(1):685. doi:10.1186/s12889-021-10708-w

40. Singh AK, Misra A. Impact of COVID-19 and comorbidities on health and economics: Focus on developing countries and India. *Diabetes Metab Syndr Clin Res Rev*. 2020;14(6):1625-1630.

41. Dugani SB, Mielke MM, Vella A. Burden and management of type 2 diabetes in rural United States. *Diabetes Metab Res Rev*. 2021;37(5):e3410.

42. Ikponmwosa OM, Akudo ML. Effect of COVID-19 induced lockdown on diabetic patients in General Hospital Abaji-Abuja. *J Pharm Allied Sci*. 2021;18(4):3537-3546.

43. Ahmed SAKS, Ajisola M, Azeem K, et al. Impact of the societal response to COVID-19 on access to healthcare for non-COVID-19 health issues in slum communities of Bangladesh, Kenya, Nigeria and Pakistan: results of pre-COVID and COVID-19 lockdown stakeholder engagements. *BMJ Glob Heal*. 2020;5(8):e003042. doi:10.1136/bmjgh-2020-003042

44. Wambua S, Malla L, Mbevi G, et al. Quantifying the indirect impact of COVID-19 pandemic on utilisation of outpatient and immunisation services in Kenya: a longitudinal study using interrupted time series analysis. *BMJ Open*. 2022;12(3):e055815. doi:10.1136/bmjopen-2021-055815

45. Haider N, Osman AY, Gadzekpo A, et al. Lockdown measures in response to COVID-19 in nine sub-Saharan African countries. *BMJ Glob Heal*. 2020;5(10):e003319. doi:10.1136/bmjgh-2020-003319
